# Pathological Classification of Lateral Elbow Tendinopathy Based on Fiber Orientation, Blood Flow Velocity of Radial Recurrent Artery, and Patient-Reported Outcome Measures

**DOI:** 10.3390/jcm14196979

**Published:** 2025-10-02

**Authors:** Masahiro Ikezu, Shintarou Kudo, Kanta Yoshioka, Masazumi Hirata, Hidetoshi Hayashi

**Affiliations:** 1 Inclusive Medical Science Research Institute, Morinomiya University of Medical Sciences, Osaka 559-8611, Japan; ikezu1201@gmail.com; 2 AR-Ex Medical Research Center, Tokyo 158-0082, Japan; 3 Graduate School of Health Sciences, Morinomiya University of Medical Sciences, Osaka 559-8611, Japan; yoshioka-kanta@ar-ex.jp; 4 AR-Ex Oyamadai Orthopedic Clinic, Tokyo 158-0082, Japan; mhirata@utopia.ocn.ne.jp (M.H.); hayashi-hidetoshi@ar-ex.jp (H.H.)

**Keywords:** lateral epicondylitis, tendinopathy, lateral elbow pain, blood flow velocity, fiber orientation, ultrasonography, cluster analysis

## Abstract

**Background/Objectives:** This study aimed to establish a method for evaluating the pathology of lateral elbow tendinopathy (LET) using ultrasonography. **Methods:** The LET group consisted of 47 patients with 50 elbows, and the control group consisted of 50 healthy adults with 50 elbows. The variables used for the pathological classification of LET included the peak systolic velocity (PSV) of the radial recurrent artery (RRA), fiber orientation intensity, numeric rating scale (NRS), Disabilities of the Arm, Shoulder, and Hand (DASH) score, and duration of symptoms. Classification was performed using principal component and cluster analyses. **Results:** The PSV of the RRA was significantly higher in the LET group (19.10 ± 4.63 cm/s) than in the control group (16.04 ± 2.96 cm/s). The fiber orientation intensity was significantly lower in the LET group (1.62 ± 0.15) than in the control group (1.73 ± 0.12). LET can be classified into three clusters. Cluster 1 showed decreased fiber orientation and moderate NRS and DASH scores. Cluster 2 demonstrated increased PSV of the RRA and severe NRS and DASH scores. Cluster 3 maintained a normal PSV of the RRA and fiber orientation, with mild NRS and DASH scores. No statistically significant differences were noted in the duration of symptoms between clusters. However, symptom duration tended to be longer in Clusters 1, 2, and 3. **Conclusions:** This study suggests that LET can be classified into mild, inflammatory, and degenerative phases.

## 1. Introduction

Lateral elbow tendinopathy (LET), commonly known as tennis elbow, is a frequent musculoskeletal disorder characterized by tendinopathy of the common extensor tendon (CET) at the lateral epicondyle [1]. The prevalence of LET is approximately 1.3%, and it is most common in individuals aged 45–54 years [2]. Although several treatment options are available for LET, the optimal therapeutic approach remains unclear [3]. Moreover, LET has a recurrence rate of 54% within two years [3], making it a challenging condition to manage. This is partly because tendinopathy encompasses a mixture of pathologies, including disordered collagen fibers, chronic inflammation, neovascularization, synovitis, calcification, cell death, osteophyte formation, and increased intratendinous pressure [4,5,6,7], which renders its overall pathophysiology exceedingly complex. Therefore, quantitative evaluation of the pathology of LET is vital for devising treatments tailored to the underlying pathological process.

The pathology of LET can be assessed using ultrasound imaging modalities [8,9,10]. Previous studies employing ultrasound to evaluate the pathology of LET have focused on variables such as hypoechogenicity, tendon thickness, tendon tears, calcifications, bone spurs, elastography, and Doppler signal characteristics [8,9,10]. However, these ultrasonographic assessments are predominantly qualitative, with limited quantitative assessments of the pathology. Additionally, the correlation between imaging findings and specific pathologies, as well as the underlying mechanisms of pathological progression, remains poorly understood. Accurate pathological classification of LET is vital for guiding appropriate treatment strategies [3]. Previous classifications were based on surgical findings and histological analyses [1,11,12]. However, these approaches are invasive and qualitative, thereby limiting their usefulness for adequate in vivo pathological assessments. Consequently, there is a lack of quantitative, non-invasive methods for evaluating the pathology of LET.

Fourier image analysis is a technique used to quantify fiber orientation [13] and can be applied to assess tendon structure in LET. Previous studies have investigated the fiber orientation of muscles, joint capsules, and ligaments using Fourier image analysis [14,15]. Additionally, peak systolic velocity (PSV), measurable in the pulse Doppler mode, is a quantitative assessment parameter, with abnormal PSV values reportedly associated with shoulder pain [16], knee pain [17], low back pain [18], and heel pain [19]. The radial recurrent artery (RRA) supplies blood to the lateral epicondyle and synovium [20]. Therefore, evaluating the PSV of the RRA may facilitate the quantification of blood circulation in LET. Consequently, ultrasound imaging offers a non-invasive means to quantitatively assess blood flow and the degree of degeneration in tendons. The pathology of tendinopathy is thought to progress from a wavy pattern of collagen fibers, through increased neovascularization and inflammatory cell infiltration, to degeneration [1]. Based on these considerations, we hypothesized that in milder pathologies, pain intensifies with increasing blood flow velocity, followed by progressive tendon degeneration. This study aimed to establish a method for evaluating LET pathology using ultrasound images.

## 2. Materials and Methods

### 2.1. Study Design

This study was conducted with the approval of the Ethics Review Committee of Morinomiya University of Medical Sciences (No. 2023-061). The rights of the participants were explained in accordance with the Declaration of Helsinki, and written consent was obtained. Our data collection started on 29 September 2023 and ended on 31 March 2024.

### 2.2. Participants

The LET group consisted of 47 patients (50 elbows). The inclusion criteria were tenderness on the lateral side of the elbow, pain on resisted wrist extension, and LET symptoms lasting for at least three months [10]. The exclusion criteria were age < 18 years, glucocorticoid injection within the preceding three months, and previous elbow surgery [10]. The control group comprised 50 healthy adults and 50 elbows. The inclusion criteria were pain-free clinical examination of the lateral elbow, absence of tenderness in the lateral elbow, and no pain on resisted wrist extension [21]. The exclusion criteria were age < 20 years and a history of current or previous lateral elbow pain [21]. The demographic data are summarized in Table 1.

### 2.3. Ultrasonographic Assessments

Ultrasonographic assessments were conducted using a LOGIQ P10 ultrasound system (GE Healthcare, Chicago, IL, USA) equipped with a 4–15 MHz linear transducer. All measurements were performed by a single physical therapist with six years of experience in ultrasound applications. The LET group underwent measurements on the affected side, and the control group underwent measurements on the dominant hand.

**Figure 1:**
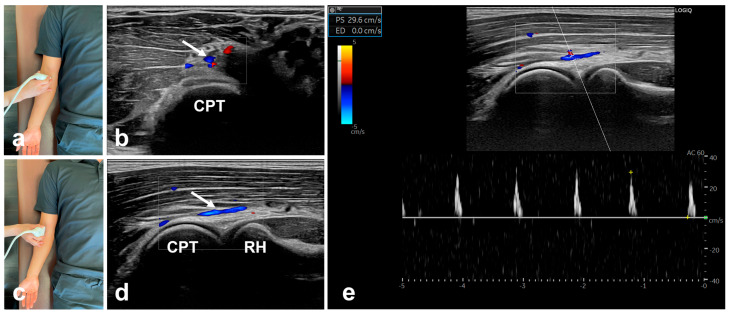
Peak systolic velocity (PSV) measurements of the radial recurrent artery (RRA). (**a**) Transducer position for short-axis view of the RRA; (**b**) Short-axis view of the RRA; the white arrow shows the RRA; (**c**) Transducer position for longitudinal-axis view of the RRA; (**d**) Longitudinal axis-view of the RRA; the white arrow shows the RRA; (**e**) Pulse Doppler sonograms of the RRA. PSV, peak systolic velocity; RRA, radial recurrent artery. CPT, capitellum of the humerus; RH, radial head.

Participants were positioned supine with the elbow fully extended (0°) and the forearm supinated to 90° for PSV measurements of the RRA. A transducer was placed in the short-axis view of the distal anterior forearm to identify the radial arteries. Based on anatomical studies, the transducer was slid proximally along the anterior forearm to locate the RRA, which branched laterally from the radial artery (Figure 1) [20]. Subsequently, the transducer was rotated by 90° to align it with the RRA and obtain a longitudinal axis view. The PSV of the RRA was assessed three times using pulse Doppler mode, and the average of the measurements was used for analysis. The Doppler incidence angle was maintained at 60° during the PSV measurements.

**Figure 2:**
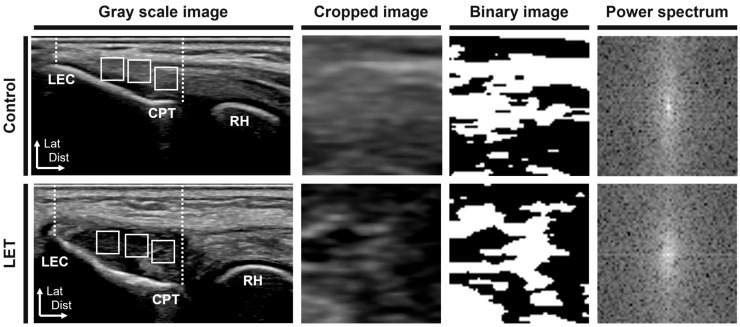
Fiber orientation measurements of the common extensor tendon on the lateral epicondyle. White dotted lines indicate the region of interest. White squares indicate the cropped area. LET, lateral elbow tendinopathy; LEC, lateral epicondyle; CPT, capitellum of the humerus; RH, radial head; Lat, lateral; Dist, distal.

The measurement positions for fiber orientation were supine with elbow flexion at 90° and forearm pronation at 90°. To visualize the CET in the longitudinal axis view, the transducer was placed over the lateral epicondyle and radial head (Figure 2, first column). The regions of interest in the image were randomly set at three locations on the CET between the lateral epicondyle and capitellum of the humerus and cropped as an 8-bit grayscale image of 64 × 64 pixels using ImageJ software 1.52a (National Institutes of Health, Bethesda, MD, USA) (Figure 2, second column). The cropped image was analyzed for fiber orientation using fiber orientation analysis software (FiberOri8single03; Tsukuba University, Ibaraki, Japan) [13]. The analysis was performed as follows. First, the cropped image was binarized (Figure 2, third column). Subsequently, the power spectrum was obtained using Fourier transformation (Figure 2, fourth column). The power spectrum was then divided into 180 regions ranging from a central angle of 0° to 180°, and the mean amplitude was obtained for each region. Each angular component indicated that a larger amplitude corresponded to a greater number of fibers oriented at that specific angle. Therefore, when the fiber arrangement is irregular, the amplitudes across all angular components tend to be similar, and the approximate ellipse approaches a circle. Conversely, when the fibers were arranged regularly, there were more fibers with specific angular components, resulting in larger amplitudes. This causes the major axis of the approximate ellipse to become longer than the minor axis. Hence, the ratio of the major axis to the minor axis of the approximate ellipse serves as a measure of the fiber orientation intensity (≥1).

### 2.4. Patient-Reported Outcome Measures

Patient-reported outcome measures included the numeric rating scale (NRS) and Disabilities of the Arm, Shoulder, and Hand (DASH) scores.

### 2.5. Intra-Rater Reliability

Participants included five healthy adults with 10 elbows (four males and one female; mean age, 24.60 ± 1.50 years; height, 167.40 ± 8.33 cm; and weight, 61.20 ± 11.37 kg) without a history of elbow joint disease. Ultrasonographic assessments of fiber orientation and PSV of the RRA were conducted using the methodology described in Section 2.3. Retests were carried out at intervals of three to seven days.

### 2.6. Statistical Analysis

The reliability of the ultrasonographic assessments was evaluated using the intraclass correlation coefficient (ICC) (1,3). The criteria for ICC were defined as follows [22]: <0.00 = poor, 0.00–0.20 = slight, 0.21–0.40 = fair, 0.41–0.60 = moderate, 0.61–0.80 = substantial, and 0.81–1.00 = almost perfect. The minimal detectable change at the 95% confidence interval (CI) (MDC95%) was defined as follows [23]:

MDC95%=1.96×√2×SEM

where SEM is the standard error of measurement and is calculated as:

SEM=SD√(1−ICC)

The PSV and fiber orientation intensity between the LET and control groups were compared using unpaired t-tests. Principal component (PC) and cluster analyses were performed to classify the pathology of LET. In the PC analysis, PSV, fiber orientation intensity, NRS, DASH score, and symptom duration in the LET group were used as variables and were then integrated into the composite variables. The validity of the PC analysis was verified using Bartlett’s sphericity test and the Kaiser–Meyer–Olkin test. The criteria used for the PC analysis included an eigenvalue of ≥1.00. Hierarchical cluster analysis using Ward’s linkage method was performed on the PC scores of the LET group. The number of clusters was determined based on the variation in the coefficients of the cluster aggregation process. One-way analysis of variance was performed to compare the demographic information, PSV of the RRA, fiber orientation intensity, NRS, and DASH scores between the clusters. Post hoc tests were performed using Tukey’s test. All statistical analyses were performed using SPSS software (version 27.0; IBM Corp., Armonk, NY, USA). The significance level was set at 5%.

## 3. Results

### 3.1. Intra-Rater Reliability

The results of the intra-rater reliability (ICC 1, 3) for the PSV of the RRA and fiber orientation intensity are shown in Table 2. In this study, ultrasonographic assessments showed almost perfect reliability according to the criteria of Landis and Koch [22].

### 3.2. Ultrasonography Assessments

**Figure 3:**
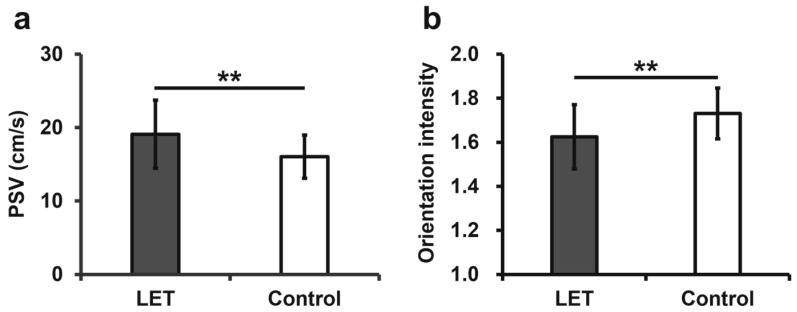
Comparison of the ultrasonographic assessments between the LET and control groups. (**a**) Comparison of PSV between the LET and control groups; (**b**) Comparison of orientation intensity between the LET and control groups. LET, lateral elbow tendinopathy; PSV, peak systolic velocity; ** *p* < 0.01.

The results for the PSV of the RRA and the fiber orientation intensity are shown in Figure 3. The mean PSV of the RRA was 19.10 ± 4.63 cm/s in the LET group and 16.04 ± 2.96 cm/s in the control group. The PSV of the RRA was significantly higher in the LET group than in the control group (*p* < 0.01). The mean fiber orientation intensity was 1.62 ± 0.15 and 1.73 ± 0.12 in the LET and control groups, respectively. The fiber orientation intensity was significantly lower in the LET group than in the control group (*p* < 0.01).

### 3.3. Principal Component Analysis

The results of Bartlett’s sphericity test were significant (*p* < 0.01), and the Kaiser–Meyer–Olkin test was 0.575, confirming the validity of the PC analysis. The PCs were consolidated into two groups (Table 3) based on the results of the PC analysis. The primary variables constituting PC1 were NRS, PSV, and DASH scores. The primary variables constituting PC2 were symptom duration and fiber orientation intensity. The principal component score–coefficient matrix is presented in Table 4.

### 3.4. Cluster Analysis

**Figure 4:**
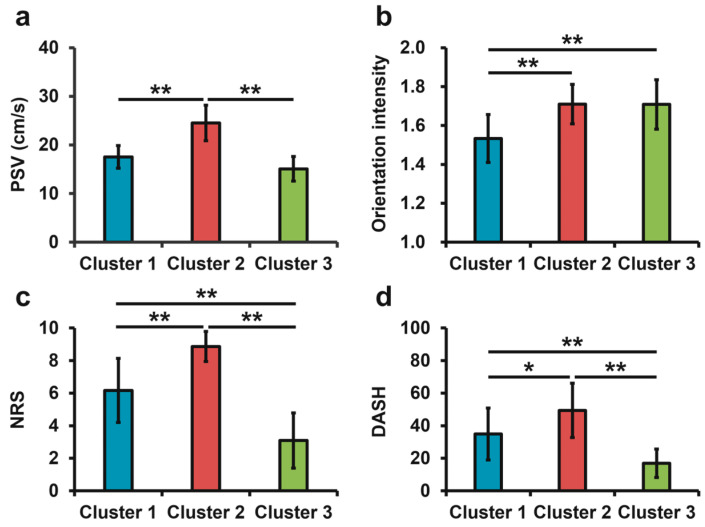
Ultrasonographic assessments and patient-reported outcome measures in each cluster. (**a**) Comparison of PSV among clusters. (**b**) Comparison of fiber orientation intensity among clusters. (**c**) Comparison of NRS among clusters. (**d**) Comparison of DASH among clusters. PSV, peak systolic velocity; NRS, numerical rating scale; DASH, Disability of the Arm, Shoulder, and Hand; * *p* < 0.05; ** *p* < 0.01.

The results of the hierarchical cluster analysis showed that the data could be classified into three clusters. No statistically significant differences were observed in the demographic characteristics of the clusters (Table 5). However, the duration of symptoms tended to be longer in the sequence of Clusters 1, 2, and 3 (*p* = 0.07). Comparisons of ultrasonographic assessments and patient-reported outcome measures for each cluster are shown in Figure 4. The mean PSV of the RRA was 17.55 ± 2.32 cm/s, 24.51 ± 3.66 cm/s, and 15.09 ± 2.54 cm/s for Cluster 1, 2, and 3, respectively. PSV was significantly higher in Cluster 2 than in Clusters 1 and 3 (Cluster 1 vs. Cluster 2, *p* < 0.01; Cluster 2 vs. Cluster 3, *p* < 0.01) (Figure 4a). No significant difference was observed between Clusters 1 and 3 (*p* = 0.05). The mean fiber orientation intensity was 1.53 ± 0.12 for Cluster 1, 1.71 ± 0.10 for Cluster 2, and 1.71 ± 0.13 for Cluster 3. The fiber orientation intensity was significantly lower in Cluster 1 than in Clusters 2 and 3 (Cluster 1 vs. Cluster 2, *p* < 0.01; Cluster 1 vs. Cluster 3, *p* < 0.01) (Figure 4b). No significant difference was observed between Clusters 2 and 3 (*p* = 1.00). The mean NRS values were 6.17 ± 1.97 for Cluster 1, 8.87 ± 0.92 for Cluster 2, and 3.09 ± 1.70 for Cluster 3. The NRS was highest in Cluster 2, followed by Cluster 1 and 3 (Cluster 1 vs. Cluster 2, *p* < 0.01; Cluster 1 vs. Cluster 3, *p* < 0.01; Cluster 2 vs. Cluster 3, *p* < 0.01) (Figure 4c). The mean DASH score was 34.94 ± 15.97 for Cluster 1, 49.42 ± 16.66 for Cluster 2, and 16.91 ± 8.69 for Cluster 3. DASH scores were the highest in Cluster 2, followed by Clusters 1 and 3 (Cluster 1 vs. Cluster 2, *p* = 0.01; Cluster 1 vs. Cluster 3, *p* < 0.01; Cluster 2 vs. Cluster 3, *p* < 0.01) (Figure 4d). The mean PC scores for each cluster are presented in Table 6.

## 4. Discussion

The results of this study showed that LET is associated with a decrease in fiber orientation and an increase in the PSV of the RRA. Cluster analysis demonstrated decreased fiber orientation in Cluster 1 and increased PSV of the RRA in Cluster 2. Cluster 3 maintained normal fiber orientation and PSV of the RRA. No statistically significant differences in symptom duration were detected among the clusters; however, Clusters 1, 2, and 3 tended to have longer duration.

Traditionally, the pathological classification of LET has relied on surgical findings and histological analysis [1,11,12]. Based on histological studies, Bhabra et al. proposed the following mechanism for the progression of LET pathology [1].

- Grade 1: Wave-like pattern of collagen fibers.
- Grade 2: Angiofibroblastic hyperplasia, cell hyperplasia, rounding of nuclei, disorganized collagen fibers, neovascularity.
- Grade 3: Cell depletion, matrix breakdown, collagen discontinuity, and small particle tears.
- Grade 4: Macroscopic tears (bone tendon separation).

However, this pathological classification system lacks the capacity for a quantitative in vivo assessment. Based on the results of the cluster analysis in this study, it is inferred that the pathology of LET progresses from an initial mild phase to an inflammatory phase, characterized by severe pain and increased blood flow, and subsequently to a degenerative phase characterized by tissue degeneration (Table 7). Based on Bhabra classification [1], it may be hypothesized that the mild phase corresponds to Grade 1, the inflammatory phase to Grade 2, and the degenerative phase to Grade 3. Therefore, this study suggests the possible delineation of three LET pathologies based on a non-invasive quantitative evaluation of fiber orientation, PSV of the RRA, and patient-reported outcome measures.

LET is reportedly associated with disorganized collagen fibers [1,24]. Traditionally, collagen fiber orientation has been quantified using polarized light imaging [25], second-harmonic generation microscopy [26], and ultrasonographic tissue characterization [27]. However, these methodologies are invasive and require specialized equipment, thereby limiting their feasibility in clinical settings. The low fiber orientation intensity determined by Fourier image analysis indicated an irregular fiber orientation [13]. Therefore, low fiber orientation is thought to reflect Grade 3 pathology according to the Bhabra classification [1]. LET is also associated with increased neovascularization [1,8,9,10]. The mechanism of LET is thought to involve increased compressive stress at the origin of CET [28]. Excessive compressive stress enhances the synthesis of glycosaminoglycans and proteoglycans, leading to an increase in intratendinous pressure [5]. Subsequently, increased intratendinous pressure induces hypoxia within the tendon, which in turn promotes neovascularization [5,29]. Embolization of neovascularization in LET reportedly improves pain and patient-reported outcome measures [30]. Therefore, an increase in RRA blood flow velocity is thought to reflect neovascularization. Moreover, some cases of LET are accompanied by synovitis [6]. A study of rotator cuff tears reported a correlation between the PSV of the anterior humeral circumflex artery and synovitis severity [16]. Given that the RRA supplies blood not only to the lateral epicondyle but also to the synovium [20], increased PSV of the RRA in the LET group may also reflect synovitis. However, the PSV of the RRA alone cannot clearly determine whether it reflects neovascularization or synovitis. Future studies incorporating histological analyses will be necessary.

Various treatment methods for LET have been reported, including physical therapy, extracorporeal shock wave therapy, prolotherapy, transcatheter arterial embolization, platelet-rich plasma injections, and percutaneous ultrasonic tenotomy [30,31,32,33,34,35]. However, the most effective treatment modality has not yet been established [3]. The findings of this study suggest that noninvasive, quantitative, and in vivo classification of LET pathology may underpin treatments tailored to specific pathological features.

This study had some limitations. First, this was a cross-sectional study. In the future, it will be necessary to investigate the treatment outcomes in each cluster. Second, we were unable to clarify the validity of ultrasonographic assessments of RRA. However, we believe that we were able to appropriately assess the PSV of the RRA because we estimated its course based on an anatomical study [20], and the PSV of the RRA was highly reproducible. Third, this study evaluated only the blood flow velocity of the RRA. The blood supply to the lateral epicondyle involves not only the RRA but also the radial collateral artery, middle collateral artery, and interosseous recurrent artery [20]. Future studies should consider a comprehensive evaluation that includes multiple arteries. Fourth, potential confounding factors influencing PSV, such as blood pressure, vessel diameter, and autonomic nervous system activity, were not evaluated in this study. Nevertheless, many previous studies have consistently reported an association between PSV and pain [16,17,18,19]. Therefore, we believe that the evaluation of the PSV of the RRA in LET has significant clinical implications. Fifth, all measurements were performed by a single physical therapist. However, this approach may have introduced bias, and future studies involving multiple examiners are warranted to confirm the generalizability of our findings. Sixth, this study employed a small sample size to assess intra-rater reliability. Future studies with a larger sample size are needed to verify the reproducibility of these assessments. Finally, the KMO value in the PCA was low. This study found that cluster classification using ultrasound imaging yielded useful results; however, it did not reach conclusions regarding the calculation of cutoff values. This is considered a result influenced by a low KMO value, which may vary depending on sample size and the number of outcomes, necessitating further investigation.

## 5. Conclusions

This study suggests that noninvasive and quantitative evaluation of fiber orientation and PSV of the RRA can be classified into three pathologies of LET (mild, inflammatory, and degenerative). This study may contribute to developing treatment strategies tailored to the pathologies of LET. Future research necessitates further investigation of the treatment outcomes for each LET cluster.

## Figures and Tables

**Table 1:** Demographic data of participants in the lateral elbow tendinopathy (LET) and control groups.

	LET Group (50 Elbows)	Control Group (50 Elbows)	*p* Value
Age (years)	53.64 ± 8.54	49.50 ± 14.77	0.09
Sex			0.13
Male	21	28	0.16
Female	29	22	
Height (cm)	165.16 ± 7.80	165.78 ± 9.30	0.72
Weight (kg)	61.26 ± 12.45	60.68 ± 11.43	0.81
BMI (kg/m^2^)	22.30 ± 3.23	21.90 ± 2.53	0.49
Duration of symptoms (months)	13.62 ± 15.65		
NRS	6.30 ± 2.62		
DASH	35.32 ± 18.75		

Data are expressed as mean ± standard deviation. LET, lateral elbow tendinopathy; BMI, body mass index; DASH, Disability of the Arm, Shoulder, and Hand.

**Table 2:** Intra-rater reliability.

	ICC (1, 3)	95%CI	SEM	MDC95%
PSV	0.822	0.461–0.952	1.824	5.055
Fiber orientation intensity	0.841	0.509–0.957	0.024	0.067

PSV, peak systolic velocity; ICC, intra-rater correlation coefficient; CI, confidence interval; SEM, standard error of measurement; MDC95%, minimal detectable change at 95% confidence interval.

**Table 3:** The eigenvalues for each principal component.

Variable	PC1	PC2
NRS	0.878	0.016
PSV	0.819	−0.071
DASH	0.750	0.374
Duration of symptoms	−0.069	0.801
Fiber orientation intensity	0.295	−0.613

PC, principal component; NRS, numerical rating scale; PSV, peak systolic velocity; DASH, Disability of the Arm, Shoulder, and Hand.

**Table 4:** The principal component score—coefficient matrix.

Variable	PC1	PC2
PSV	0.391	−0.061
Fiber orientation intensity	0.141	−0.527
NRS	0.419	0.014
Duration of symptoms	−0.033	0.689
DASH	0.358	0.321

PC, principal component; PSV, peak systolic velocity; NRS, numerical rating scale; DASH, Disability of the Arm, Shoulder, and Hand.

**Table 5:** Demographic information for each cluster.

	Cluster 1 (24 Elbows)	Cluster 2 (15 Elbows)	Cluster 3 (11 Elbows)	*p* Value
Age (years)	54.71 ± 9.73	53.73 ± 8.60	51.18 ± 5.17	0.53
Sex				0.50
Male	12	6	3	
Female	12	9	8	
Height (cm)	167.25 ± 7.94	162.80 ± 6.25	163.82 ± 8.83	0.18
Weight (kg)	63.63 ± 13.03	59.73 ± 13.31	58.18 ± 9.68	0.42
BMI (kg/m^2^)	22.59 ± 3.38	22.41 ± 3.83	21.54 ± 1.84	0.67
Duration of symptoms (months)	18.58 ± 20.38	11.07 ± 9.31	6.27 ± 4.34	0.07

Data are expressed as mean ± standard deviation. BMI, body mass index.

**Table 6:** The mean principal component scores of each cluster.

	Cluster 1	Cluster 2	Cluster 3
PC1	−0.258 ± 0.529	1.221 ± 0.406	−1.102 ± 0.543
PC2	0.561 ± 1.029	−0.236 ± 0.647	−0.901 ± 0.384

Data are expressed as mean ± standard deviation. PC, principal component.

**Table 7:** Pathological interpretation of each cluster.

	Cluster 1	Cluster 2	Cluster 3
PSV	within normal limits	severe	within normal limits
Fiber orientation intensity	severe	within normal limits	within normal limits
NRS	moderate	severe	mild
DASH	moderate	severe	mild
Duration of symptoms	long	intermediate	short
Phase	degeneration phase	inflammatory phase	mild phase

PSV, peak systolic velocity; NRS, numerical rating scale; DASH, Disability of the Arm, Shoulder, and Hand.

## Data Availability

The data are available upon request from the corresponding author.

## Abbreviations

LET	Lateral Elbow Tendinopathy
CET	Common Extensor Tendon
PSV	Peak Systolic Velocity
RRA	Radial Recurrent Artery
DASH	Disabilities of the Arm, Shoulder, and Hand (score)
NRS	Numeric Rating Scale (for pain)
ICC	Intraclass Correlation Coefficient
MDC95%	Minimal Detectable Change at the 95% Confidence Interval
PC/PC1/PC2	Principal Component(s)
